# Can attention-deficit/hyperactivity disorder be considered a form of cerebellar dysfunction?

**DOI:** 10.3389/fnins.2025.1453025

**Published:** 2025-01-22

**Authors:** Valeria Isaac, Vladimir Lopez, Maria Josefina Escobar

**Affiliations:** ^1^Center for Social and Cognitive Neuroscience, School of Psychology, Universidad Adolfo Ibáñez, Santiago, Chile; ^2^Escuela de Psicología, Facultad de Ciencias Sociales, Pontificia Universidad Católica de Chile, Santiago, Chile

**Keywords:** attention-deficit/hyperactivity disorder, development, motor impairment, cerebellar dysfunction, cortico-cerebellar network

## Abstract

Attention-deficit/hyperactivity disorder (ADHD) is a heterogenous disorder, commonly described for presenting difficulties in sustained attention, response inhibition, and organizing goal-oriented behaviors. However, along with its traditionally described executive dysfunction, more than half of the children diagnosed with ADHD have been reported to show difficulties with gross and fine motor skills, albeit motor impairments in ADHD continue to be a neglected area of clinical attention. The rapidly growing field of the clinical cognitive neuroscience of the cerebellum has begun to relate cerebro-cerebellar circuits to neurodevelopmental disorders. While the cerebellum’s role in motor function, such as balance, motor coordination, and execution, is well recognized, ongoing research has evidenced its additional and fundamental role in neurocognitive development and executive function, including attention and social cognition, which are all areas of impairment commonly found in ADHD. Interestingly, neuroimaging studies have consistently shown differences in cerebellar volume and functional connectivity between ADHD and typically developing children. Furthermore, methylphenidate is known to act at the cerebellar level, as intrinsic cerebellar dopaminergic systems involved in attention and motor function have been identified. This article reviews some of the main findings linking cerebellar dysfunction to ADHD behavioral symptoms and incorporates the cerebellum as a possible neurological basis and differentiating indicator within the condition. We suggest considering more rigorous assessments in future ADHD studies, including cerebellar-associated skill evaluations to correlate with symptom severity and other detected outcomes, such as executive dysfunction, and study possible associative patterns that may serve as more objective measures for this diagnosis.

## 1 Introduction

Attention-deficit/hyperactivity disorder (ADHD) is one of the most prevalent conditions in pediatric psychopathology, estimated to affect approximately 3 to 9.5% of the global population (Polanczyk et al., 2007; Thomas et al., 2015), predominantly affecting school-aged children. Its characteristic symptoms, inattention, hyperactivity, and impulsivity (American Psychiatric Association, 2022) are often explained due to impairments in neuropsychological executive functions such as response inhibition, working memory, and inhibitory control (Barkley, 1997; Martinussen et al., 2005; Willcutt et al., 2005). Another prominent feature of ADHD is that it is often accompanied by a high percentage of comorbid disorders across the lifespan. In adulthood, between 65 and 89% of patients with ADHD suffer from one or more additional psychiatric disorders, such as mood and anxiety disorders, substance use disorders, and personality disorders (Sobanski, 2006). In younger populations, ADHD is frequently associated with psychiatric comorbidities, including learning disorders, conduct disorders, anxiety, and depression (Kessler et al., 2006). ADHD has been frequently associated with motor deficiencies, with 45–70% of children diagnosed with ADHD reported to show difficulties in both gross and fine motor skills (Kaiser et al., 2015; Pitcher et al., 2003; Sayal et al., 2018). Balance and bilateral coordination (including manual dexterity) are the most commonly reported areas of motor function impairment (Buderath et al., 2009; Fliers et al., 2008; Ghanizadeh, 2010; Goulardins et al., 2013; Karaokur et al., 2017; Konicarova et al., 2014).

Historically, ADHD has been associated with disturbances in the maturation of executive function (EF) due to frontostriatal and frontoparietal dopamine-signaling deficits (Aboitiz et al., 2014), while motor disturbances were not initially considered part of its diagnostic criteria. Past hypotheses explaining the motor difficulties often found in individuals with ADHD have taken two main approaches: The first attributes these motor difficulties to the basic symptomatic triad of ADHD, influenced by inattention (Ghanizadeh, 2010) and/or impulsivity (Tseng et al., 2004). The severity of inattentiveness score has been found to correlate with and predict poor motor performance, such as coordination problems (Fliers et al., 2008), as well as deficits in fine motor skills or writing (Ghanizadeh, 2010; Kaiser et al., 2015). Piek et al. (2004) explored the link between inattention, motor ability, and aspects of EF in children with ADHD (6–15 years of age) and reported strong correlations between poor motor coordination and inattention but weaker correlations with EF, despite the fact that research has established an undisputed association between EF and ADHD (Willcutt et al., 2005). Tseng et al. (2004) found that measures of sustained attention, along with additional impulse control, were good concurrent predictors of fine and gross motor skills in 6- to 11-year-olds with ADHD, with attention and impulse control being the two best predictors of fine motor skill performance.

The second hypothesis explaining motor problems in ADHD suggests these may be secondary to a comorbidity, particularly developmental coordination disorder (DCD), defined as severe impairment in learning and execution of coordinated motor skills (American Psychiatric Association, 2022), where an estimated 50% of children diagnosed with ADHD have been found to meet the criteria for DCD (Montes-Montes et al., 2021). Notwithstanding, even when ADHD children do not meet the criteria of DCD, they continue to show weaker motor skills than typically developing children when evaluated (Klupp et al., 2021; Manicolo et al., 2016; Poblano et al., 2014).

A few decades ago, a line of research emerged suggesting cerebellar dysfunction to ADHD symptoms after Castellanos et al. reported significantly smaller cerebellar hemispheric volumes in ADHD children using quantitative brain magnetic resonance imaging (Castellanos, 2002; Castellanos et al., 1996). Following neuroimaging studies describing structural differences in the brain of ADHD children when compared to typical peers continued to establish atypical cerebellar volume as one of the most consistent findings in the neuroimaging literature on ADHD (Pereira-Sanchez and Castellanos, 2021; Valera et al., 2007) particularly with gray matter reductions in the posterior-inferior lobules, VIII to X (Berquin et al., 1998; Bledsoe et al., 2011; Mostofsky et al., 1998), and functional cerebellar dysconnectivity (Feng et al., 2023; Tomasi and Volkow, 2012; Wang et al., 2022).

The cerebellum is considered a vital component in the human brain that has traditionally been studied for its central role in motor movement regulation and balance control. It is known to integrate different sensory inputs such as visual, vestibular, and somatosensory information responsible for motor coordinated movements, postural control, and the planning of sequenced actions (Horak and Macpherson, 1996), contributing to the quality of movement in conjunction with the prefrontal cortex (PFC) and basal ganglia (Welniarz et al., 2021). The cerebellum is also known to play a pivotal role in fine motor such as bilateral manual dexterity (Soteropoulos and Baker, 2008), speech articulation (Salman and Tsai, 2016), and the realization of coordinated eye movements including the control of fixed eye movements through projections from the frontal eye field (Pretegiani et al., 2018). While the cerebellum is often described as a structure mainly involved in motor coordination, it has a well-established role in numerous facets of cognitive, emotional, and behavioral functioning (Koziol et al., 2014) which are all areas reported as dysfunctional in ADHD. To date, there is enough evidence to sustain that the cerebellum could be playing a central role in ADHD outcomes and symptomatic behaviors, particularly in children. In this study, we will review some main findings that link the cerebellum directly to all symptoms commonly reported in ADHD.

## 2 Evidence for cerebellar involvement in all reported ADHD symptoms

### 2.1 Cognitive performance and executive functioning

One of the most currently accepted hypotheses of the underlying physiopathology in ADHD is the deficit in dopamine-signaling mechanisms, associated with genetic factors encoding for dopamine receptor DRD4 and dopamine transporter DAT1, affecting PFC, basal ganglia, thalamus, and amygdala circuits, which participate in EF, suggesting a reduction of executive control associated with abnormalities in frontoparietal and frontostriatal network function (Antshel et al., 2014) which would directly impact working memory, the ability to sustain attention to goal-directed tasks, and self-regulate social–emotional behaviors. Studies using neuropsychological measures reported that overall cognitive abilities are significantly lower among ADHD subjects, including significant impairment in all EF tasks (Frazier et al., 2004).

While the cerebellum’s role in motor function is well recognized, a substantial portion of the cerebellum is associated with non-motor functions connected to different large-scale networks in the neocortex, including frontal and parietal regions typically involved in high-order cognitive processing, providing the means through which association areas and the cerebellum may influence each other’s operation (Ramnani, 2012). Resting-state functional connectivity MRI studies show that cerebral networks involved in movement, attention, and limbic valence, as well as frontoparietal and default systems associated with creativity and imagination, map onto the cerebellum with topographic specificity (Buckner et al., 2011). The dorsal attention, ventral attention, frontoparietal, default mode, and salience networks map onto focal areas within the posterior lobe of the cerebellum. Lobules VI and Crus I are engaged in language and verbal working memory; lobule VI in spatial tasks; lobules VI, Crus I, and VIIB are activated by executive functions such as working memory, planning, organizing, and strategy formation; and vermal lobules VI and VII are involved in emotional processing. Language is heavily right lateralized and spatial functions left-lateralized, reflecting crossed cerebro-cerebellar projections (Schmahmann, 2019). Studies have demonstrated the close relationship between cerebellar function and working memory (Stein, 2021), attention modulation (Mannarelli et al., 2019), and higher order executive functioning (Pretegiani et al., 2018) through cortico-cerebellar circuits (Zhu et al., 2023), including bilateral projections between the neocerebellum and dorsolateral PFC (Habas, 2021) as well as the basal ganglia (Caligiore et al., 2017). These cortico-cerebellar networks have been described as critical during participation in tasks that are difficult and novel, especially those requiring unpredictable and quick adjustments, demanding sustained attention (Diamond, 2000), directed visual attention and working memory processes (Brissenden and Somers, 2019), and during social interactions (Li et al., 2023).

Studies with patients with cerebellar lesions have been found to show deficits in the ability to shift attention (Akshoomoff and Courchesne, 1994) and direct goal-oriented behavior (Beuriat et al., 2022; Botez et al., 1989). Cerebellar symptoms have also been associated with difficulties in spatial working memory in children and adolescents with ADHD (Ferrin and Vance, 2012). Therefore, the cortico-cerebellar network allows the cerebellum not only to contribute to the coordination of movements, as once thought, but also to the modulation and integration of all higher cortical functions, including those compromised in ADHD.

### 2.2 Motor performance

Children with ADHD have consistently shown poor performance on motor skill tests when compared to typically developing children (Brossard-Racine et al., 2012; Goulardins et al., 2013), particularly in activities requiring fluent coordinated movements (Fliers et al., 2008; Kaiser et al., 2015), handwriting (Brossard-Racine et al., 2011), bimanual coordination (Piek et al., 1999), and gait, balance, and postural control (Buderath et al., 2009; Mao et al., 2014; Simmons et al., 2020). All of these areas of motor performance are directly associated with cerebellar function. Even DCD, a common comorbidity to ADHD, that specifically impairs a child’s ability to coordinate movement and learn motor skills to perform everyday activities, has also been associated with cerebellar dysfunction (Gill et al., 2022; Mariën et al., 2010). The cerebellum has been considered essential during motor learning and is strongly implicated in detecting and correcting motor errors (Popa et al., 2016). These motor errors are defined as the discrepancy between the predicted consequences of motor commands and the sensory feedback, which are encoded in the cerebellar cortex in the context of a forward internal model that generates predictions about the upcoming movement and drives learning and adaptation. Cerebellar Purkinje cells have been found not only to drive motor learning but also to expand the capacity of motor circuits (Nguyen-Vu et al., 2013).

The cerebellum is responsible for the complex integration of multiple postural information coming from vestibular, visual, and proprioceptive inputs. Cerebellar circuitry regulates static and dynamic parameters playing a key role in establishing balance stability and steady gait (Dijkstra et al., 2020). Postural instability and sway abnormalities are commonly reported in children with ADHD, and studies have previously shown a relationship between postural control alterations and the cerebellum in ADHD (Hove et al., 2015). During static balance assessments, ADHD-diagnosed children show impaired postural control evidenced in an increased sway of their center of pressure (Cheng and Wang, 2007) and significantly smaller loss of stability area (Isaac et al., 2017) than their typical peers. In dynamic motor control, existing evidence in relation to gait (Bucci et al., 2016; Buderath et al., 2009; Kim et al., 2017; Shum and Pang, 2009) shows that children with ADHD walk with less consistency of speed (Meachon et al., 2023), less rhythmic and less automatic gait (Leitner et al., 2007), greater variability of cadence and step time, and greater variability for stride length when walking at an increased stepping rate (Simmons et al., 2020).

Cerebellar involvement in complex manual tasks has also been documented (Kühn et al., 2012; Rau et al., 2024), including studies of neural correlates in handwriting during childhood (Palmis et al., 2021; Zhang et al., 2022). Regarding upper extremity motor control, children with ADHD have been found to show less fluidity in arm movement (Van and Thomas, 2002), reduced coordination in manual dexterity (Brossard-Racine et al., 2012), and slower performance in fine motor tasks (Scharoun et al., 2013). Studies further find that children with ADHD have less legible handwriting (Mokobane et al., 2019; Shen et al., 2012), with a tendency to be less accurate in keeping letter size within boundaries, spacing, and alignment (Tucha and Lange, 2001).

In addition to gross and fine motor, poor eye movement performance has frequently been reported in children with ADHD (Goto et al., 2010) when compared to typically developing children. Children with ADHD show a larger number of errors during anti-saccade tasks as well as frequent intrusive saccades during smooth pursuit and fixation (Fried et al., 2014; Maron et al., 2021), less precise and stable movements during visual tracking (Munoz et al., 2003; Slaats-Willemse et al., 2005), and need more time to complete oculomotor tasks than those without ADHD (Sherigar et al., 2023). The cerebellum, in particular, is a structure of utmost importance in optimizing goal-directed eye movements such as saccadic and smooth pursuit eye movements to stabilize the images of moving targets (Prsa and Their, 2022). Animal and human studies evidenced that damage to specific parts of the cerebellum causes imprecise visually guided eye movements and smooth pursuit deficits, such as those of the flocculus/paraflocculus or those of the vermis including lobule VI, VII, the uvula, and the deep cerebellar nuclei (Baier et al., 2009).

Timing of motor actions is another aspect that has been found to be impaired in ADHD (Zelaznik et al., 2012), showing slower timing of movements and greater variability between sequences of actions (Chen et al., 2013; Noreika et al., 2013; Shiels Rosch et al., 2013; Slater and Tate, 2018), especially when movements require more planning and complex motor coordination (Kaiser et al., 2015). Along with the motor cortices and basal ganglia, the cerebellum plays a key role in regulating the timing of self-initiated movements and predicting the timing of periodic events (Tanaka et al., 2021) which allows for the execution of synchronized movements such as spontaneous dance and clapping to music (Bo et al., 2008). The cerebellum appears to enable these controls by linking sensory inputs and motor timing, particularly in the range from a few hundred milliseconds to a second (Mauk and Buonomano, 2004). Abnormalities in reproducing temporal durations, with intervals as brief as 400 ms, have been documented in children and adolescents with ADHD, showing impairments in duration discrimination, and the precision and reliability with which they reproduced the intervals (Toplak et al., 2006). Castellanos and Tannock (2002) proposed a temporal processing candidate endophenotype for ADHD linked to deficits in time estimation, emphasizing that intra-individual variability in task performance is “the most striking clinical characteristic in ADHD.” These authors further state that temporal variability in symptom expression in ADHD is linked to cerebellar dysfunction. Timing deficits in ADHD are furthermore supported by functional neuroimaging studies that show dysfunctions in the key inferior fronto-striato-cerebellar and frontoparietal networks that mediate timing functions (Noreika et al., 2013).

### 2.3 Social cognition

In addition to executive dysfunction and motor impairment, it is widely acknowledged that social functioning and social cognition are also altered in children (Arango-Tobón et al., 2023) and adults with ADHD (Morellini et al., 2022). These alterations often lead to socially inappropriate responses, resulting in alterations in their adaptive behavior. Social cognition impairments in ADHD have been observed in the domains of theory of mind (ToM) (Parke et al., 2021), demonstrated by inferior performance in understanding intentional behaviors (Mary et al., 2016; Mohammadzadeh et al., 2016), empathy and emotional intelligence. Children with ADHD have shown poorer performance in tasks measuring emotional prosody (especially in perceiving emotionally angry statements) and intermodal skills (Kis et al., 2017; Uekermann et al., 2010), as well as recognizing facial emotions, with the most significant impairments observed in negative emotions such as anger and fear (Bora and Pantelis, 2016).

Schmahmann and Sherman (1998) introduced the cerebellar cognitive affective syndrome (CCAS), characterized by disturbances in executive function, visual–spatial processing, linguistic skills, and regulation of affect, linking this syndrome with cerebellar posterior lobe lesions (Schmahmann, 2019). Since then, studies evidencing cerebellar involvement in the regulation of affective reactions as well as in forming the association between sensory stimuli and their emotional values have increased to the point where cerebellar functional topography for emotional and social processing has become well-established (D’Agata and Orsi, 2022).

The cerebellar vermis, which is the principal target of limbic connections (Berntson and Torello, 1982), has been implicated in the modulation of emotions (Stoodley and Schmahmann, 2010), and lobules VI and VII (crus I–II), in the posterolateral cerebellar hemisphere, have been implicated in social cognition that involves more complex cognitive processes such as empathy (Sokolov, 2018). An increasing amount of evidence has confirmed that the cerebellum is actively involved in emotional recognition specifically processing facial expressions of negative emotion (Ferrucci et al., 2012), particularly in fear-related processes, possibly due to reciprocal cerebellar connections with the amygdala (Turner et al., 2007).

Recent studies have highlighted the function of the cerebellum in supporting social cognition and interaction, through function-specific mentalizing and somatomotor processes (Van Overwalle et al., 2015) using encoded internal models that reproduce the dynamic properties of the body involving sequencing and planning of action, to automate and fine-tune not only voluntary motor processes but also cognitive processes (Ito, 2008) in collaboration with the cortex, such as related ToM processes based on the prediction of sequential events. A previous meta-analysis by Van Overwalle et al. (2014) of fMRI studies found robust clusters of activation in the cerebellum recruited during social-cognitive processes that overlap with cerebellar non-social functions such as motor functions, emotions, executive control, and language. Therefore, in addition to balance and motor coordination functions, non-motor behaviors, including cognition and social behavior, appear to be equally affected by deficits in cerebellar networks.

### 2.4 Autonomic and arousal regulation

One theoretical model developed to explain ADHD etiopathogenesis is the state-regulation theory introduced by Sanders (1983), which suggests that a decreased ability to regulate brain arousal may contribute to the higher-level cognitive deficits in ADHD. Arousal state regulation in the brain is related to the release of the catecholamine norepinephrine (NE) and dopamine in the PFC (Arnsten and Li, 2004). NE has marked effects on PFC function, and small changes in the autonomic arousal systems can markedly alter the connectivity of PFC networks impacting EF and cognitive performance (Arnsten and Pliszka, 2011). Autonomic dysregulated functions would directly impact brain arousal through locus coeruleus (LC) connectivity and NE pathways, coordinated by the central autonomic network (CAN), altering the overall efficiency of cognitive performance (Diamond, 2005) leading to both executive dysfunction and hyperactivity symptoms (Sergeant, 2000). Neurophysiological studies measuring activity from the autonomic sympathetic nervous system (such as heart rate, electrodermal responses, and pupillary dilatation) have evidenced dysfunction in individuals with ADHD, more often in the direction of sympathetic hypo-arousal than hyper-arousal, particularly at rest and during tasks requiring response regulation and sustained attention (Bellato et al., 2020).

Cerebellar structures were associated with autonomic cardiovascular functions by early experiments, which showed that stimulation of the cerebellar vermis interferes with autonomic reactions, by limiting blood pressure oscillations and inducing hyperventilation (Moruzzi, 1940; Nisimaru, 2004). Further studies found anatomical connections between the fastigial nucleus and the hypothalamus suggesting cerebellar vermis involvement in autonomic and emotional regulatory functions (Snider, 1950). Later studies confirmed the existence of a prominent NE pathway from the brainstem nucleus LC to all regions of the cerebellar cortex and deep cerebellar nuclei (Olson and Fuxe, 1971), recognizing the cerebellum as a key region of the CAN (Benarroch, 1993) and demonstrating neuromodulatory effects of NE on the spontaneous and evoked discharge of Purkinje neurons (Freedman et al., 1977). NE is capable of producing a relative or absolute enhancement of stimulus-driven activity in the primary output cells of the cerebellar cortex, whereby Purkinje cells exhibiting little or no response to peripheral stimuli become responsive to such inputs in the presence of NE (Moises et al., 1990). This evidence indicates that NE can produce a spectrum of effects on the spontaneous and evoked discharge of Purkinje neurons, all of which serve to regulate the responsiveness of these cells to synaptically driven inputs (Waterhouse and Navarra, 2019). The net outcome of LC-NE regulation of cerebellar circuit operations may be that voluntary and reflex motor activities, cognitive operations, and emotive responses are optimized with respect to ongoing goal-directed behaviors and unexpected environmental challenges (Predale et al., 2023) which allows for organized and adapted behavior in a stable and predictable way. Interestingly, one of the most striking clinical characteristics described by clinicians of ADHD in addition to the frequent lapses of attention is the high prevalence of intra-individual variability and inconsistency in performance (Castellanos and Tannock, 2002), which may possibly be related to cerebellar dysfunction in modulating emotional signals and behavior through extensive cerebello-cortical and cerebello-subcortical circuits.

Recent data are leading to profound changes in our understanding of cerebro-cerebellar circuits, which are now considered essential for the functional regulation of various neocortex areas, including PFC cognitive circuits, as well as of the hypothalamus and autonomic network, and the limbic system (Rudolph et al., 2023).

## 3 Could the cerebellum be a neurological basis for ADHD?

Considering the evidence that the cerebellum works as an integrated system with the cortex (including prefrontal and parietal areas), the basal ganglia, and the thalamus in a variety of functions—from the motor to cognitive tasks of varying complexity (Caligiore et al., 2017)—and that these areas play a critical role in attention, behavior organization, affect regulation, motor planning, and coordination (Cortese, 2012; Rudolph et al., 2023), we suggest that symptoms commonly reported in ADHD, such as executive function variability, balance and motor coordination impairments, difficulties in emotional and arousal regulation, and social adaptation problems, can largely be explained by cortico-cerebellar network deficiencies.

Furthermore, common psychiatric comorbidities associated with ADHD have also been related to cerebellar dysfunction. Anxiety disorders, being considered the most frequent and associated with worse clinical presentation of ADHD symptoms (Quenneville et al., 2022), have been linked to abnormalities in cerebellar vermis activity and vermis-amygdala connectivity (Chin and Augustine, 2023). Mood disorders and externalizing disorders, such as oppositional defiant disorder and personality disorders, are also highly prevalent comorbidities in ADHD (Katzman et al., 2017) that have been linked to cerebellar dysfunction, where studies in patients with cerebellar lesions have shown states of depression, maniac, and personality alterations (Lupo et al., 2019) suggesting that the cerebellum should be considered a key structure involved in the regulation of mood and emotion.

Another important aspect to consider is that the current DSM-5 classifies ADHD a neurodevelopmental condition (American Psychiatric Association, 2022), as its symptoms exhibit normative change over time (Martel et al., 2016). Studies show typical cortical development patterns in ADHD, but these are delayed by as much as 2–3 years, depending on the specific cortical region. This delay in both cortical thickness and surface area suggests a global perturbation in the mechanisms that guide cortical maturation (Shaw et al., 2007, 2012). Therefore, higher-order networks may not be directly impaired in ADHD but influenced by other circuits which may be altering the optimal development and performance of higher-order cognitive functions. The human brain does not mature in a uniform manner, and the cerebellum has been shown to be one of the first structures to start cellular differentiation. Between 20 and 40 weeks of gestation, the cerebellum undergoes an exuberant period of growth (Clouchoux et al., 2012), with a rapid development period in the third trimester of pregnancy and in the first postnatal year. This growth pattern strongly suggests the presence of a critical period for cerebellar development, making it especially vulnerable to genetic and environmental stressors disrupting development (Limperopoulos et al., 2005). Research findings suggest that the cerebellum plays a critical role in motor, cognitive, and social–behavioral development, possibly via modulatory effects on the developing cerebral cortex, guiding the maturation of remote non-motor neural circuitry and influencing cognitive learning (Gaiser et al., 2024). Cerebellar damage can cause a striking volume reduction in the opposite hemisphere of the cerebrum (Limperopoulos et al., 2005), suggesting that the cerebellum may have a direct influence on cortical maturation (Olson et al., 2023). These findings hint that early in development, the rapid growth of the cerebellum may be joined to PFC growth and activity and thus could be a key node in various neurodevelopmental disorders, as already suggested for autism spectrum disorder (Wang et al., 2014) and possibly for ADHD.

In clinical pharmaceutical treatment guidelines, methylphenidate hydrochloride (MPH) is recommended as a first-line medication for ADHD. The exact mechanism of action of MPH is not completely understood, but they are presumed to act through the dopaminergic and adrenergic pathways of the frontostriatal circuits in the brain by blocking the reuptake of dopamine into the presynaptic cleft (Nair and Moss Baylor, 2009). However, dopamine networks are not limited to frontostriatal networks. Along with the dense cerebellar connections to the frontal cortex and basal ganglia, an independent dopaminergic network exists within the cerebellum (Li et al., 2023), with high concentrations of dopamine transporters found particularly in the posterior-inferior vermis (Flace et al., 2021; Melchitzky and Lewis, 2000). Nonetheless, the effects of MPH modulating cerebellar function in ADHD have been documented (Czerniak et al., 2013; Inci Kenar et al., 2017), leading to suggest that behavioral and even motor modifications in children with ADHD taking MPH may be explained through outcomes that involve the cerebellum (Brossard-Racine et al., 2012; Bucci et al., 2016).

Research relating ADHD and cerebellar function so far has shown a high correlation between cerebellar volume reduction and ADHD symptoms, suggesting a specific contribution of the cerebellum in the etiology of this disorder (Stoodley, 2016). Goetz et al. (2017) associated impaired balance, reaction times, and cerebellar dysfunction in children with ADHD and found correlations between poor dynamic balance and reaction time consistency, concluding that impaired balance control and cognitive functioning might share a common dysfunction at the cerebellar level in ADHD. Similarly, Bledsoe et al. (2011) studied correlations between ADHD symptomatology and reductions in cerebellar vermis, finding that a large portion of the variance in reported ADHD behavior was predicted by reduced volume in posterior-inferior cerebellar vermis, suggesting the vermis of the cerebellum may be a specific structure related to symptoms of hyperactivity and impulsivity and in the modulation of attention.

As mentioned, several lines of research confirm the involvement of cerebellar structures and circuits in all areas of ADHD diagnostic outcomes. This new appreciation of cerebellar incorporation into circuits that subserve cognition and emotion mandates a deeper understanding of the cerebellum, beyond the motor domain, and its impact on neurodevelopmental disorders such as ADHD, providing potential for novel cerebellar-based approaches to diagnosis and therapy.

## 4 Conclusion

The ADHD is a highly complex and heterogeneous disorder in terms of its multidimensional outcomes, multi-factorial etiological risk factors, diverse neurocognitive impairments, and comorbid problems (Nigg et al., 2020). A large number of controversial results continue to exist within the study of ADHD biological and performance markers, as it is based exclusively on behavioral symptoms where the bottom-line issue of heterogeneity and variable severity has not been directly addressed.

Several lines of research confirm the involvement of the cerebellum in ADHD symptoms and behaviors, considering the cerebellum as a key factor in guiding the maturation of remote non-motor neural circuitry and influencing cognitive development. Evidence has indicated that the development of the cerebellum occurs very early in life and is more dependent upon environmental factors than most other brain regions (Giedd et al., 2007), and sensitive-period disruption of cerebellar-brain communication may have an impact on higher cognitive functions.

Future considerations should focus more specifically on the multidimensional assessment of functions associated with specific cerebellar areas or circuits through neuropsychological evaluations linked to neuroimaging studies, and assessment of functions that particularly nurture cerebellar growth such as vestibular and proprioceptive afferences, to establish correlations that might elucidate patterns of symptom expression. We suggest evaluating all areas involved in cerebellar function through motor, sensory, cognitive, and social performance, linked to neuroimaging results, to explore possible correlations that may be indicative of cerebellar-based ADHD endophenotypes and be used as indicators to monitor treatment response.

Future studies should focus on assessing the large spectrum of impaired performance areas simultaneously and search for correlated patterns of grouped symptoms that will not only complete the diagnosis but serve as a quantitative determination of endophenotypes that can lead to more targeted and efficient treatment programs.

## Data Availability

The original contributions presented in the study are included in the article/supplementary material, further inquiries can be directed to the corresponding author.
